# Supplementary material for: Microbiome Analysis of Traditional Grain Vinegar Produced under Different Fermentation Conditions in Various Regions in Korea
Source: Foods. 2022 Nov 10;11(22):3573. doi: 10.3390/foods11223573 (PMC9689881; doi:10.3390/foods11223573)
Supplement: Supplementary file 1 [file foods-11-03573-s001.zip › foods-1950501-SI.pdf]

Metagenomic Analysis of Traditional Grain Vinegar Produced under Different Fermentation Conditions in Various Regions in Korea

Haram Kong, Sun-Hee Kim, Woo-Soo Jeong, So-Young Kim and Soo-Hwan Yeo *

Fermented and Processed Food Science Division, Department of Agrofood Resources, NIAS, RDA, Wanju 55365, Korea

Supplementary files:

Table S1. Summary of metagenomics sequence assembly information

Figure S1. The phenotypic differences among the clusters

Table S1. Summary of metagenomics sequence assembly information

Feature		Data						
		CN_UR	JBG_BR	JBG1_UR	JBG2_UR	GN_BR	GB_FG	GB_UR
Total read bases (bp)		55,972,154	57,884,106	57,369,998	61,700,184	58,636,606	35,569,170	43,082,732
Total reads		185,954	192,306	190,598	204,984	194,806	118,170	143,132
GC content (%)		53.88	250,848	250,848	53.31	53.31	53.54	53.31
AT content (%)		46.12	46.14	47.38	46.69	46.69	46.46	46.69
Q20 (%)		88.89	95.27	95.24	94.99	88.97	88.33	87.67
Q30 (%)		80.32	88.89	88.81	88.32	80.34	79.19	78.35
Data pre-processing	Adapter & Primer Trimming	90,883	95,159	94,185	101,274	94,737	57,663	69,230
	Quality Filter	78,947	88,611	87,489	93,949	83,187	49,058	57,964
	denoisedFor	78,920	88,500	87,374	93,866	82,748	48,921	57,834
	denoisedRev	78,932	88,400	87,252	93,863	82,930	48,942	57,946
	mergedPair	78,893	88,042	86,441	93,746	82,232	48,868	57,781
	non-chimeric	78,295	87,902	72,045	93,106	77,945	45,096	50,290
Community richness & diversity index	ASVs	19	45	33	92	96	6	5
	Chao1	19	45	33	92	99	6	5
	Shannon	1.1299	1.4717	1.7443	1.6392	1.3721	1.4689	1.4764
	Gini-Simpson	0.5132	0.5381	0.5645	0.5609	0.5013	0.5861	0.5754
	Good's Coverage	1.0000	1.0000	1.0000	1.0000	0.9997	1.0000	1.0000

Type of sequencing: Illumina MiSeq

Total read bases: total number of bases sequenced.

Total reads: For Illumina paired-end sequencing, this value refers to the sum of read 1 and read 2.

Q20: Ratio of bases that have phred quality score of over 20.

Q30: Ratio of bases that have phred quality score of over 30.

Tools for each analysis are Cutadapt for adapter and primer trimming, and DADA2 for quality filtering, denoising, merging, and chimera removal.

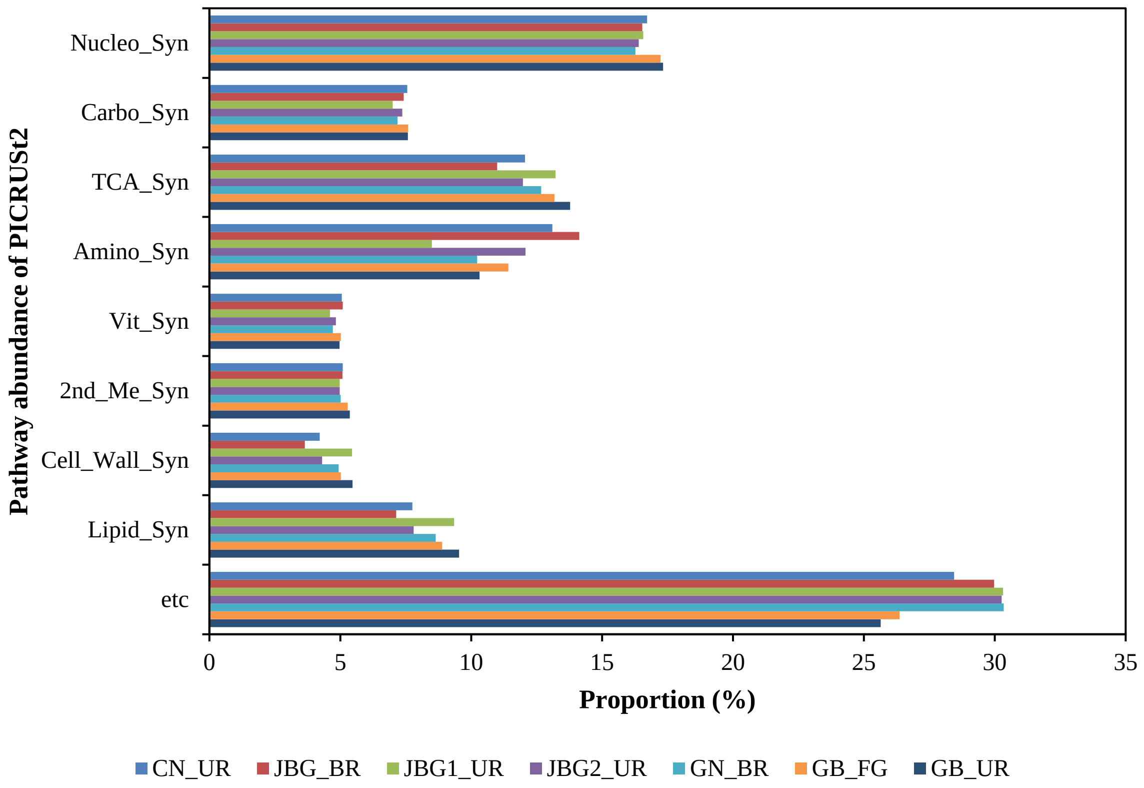

Figure S1. The phenotypic differences among the cluster
